# Wi-fi related radiofrequency electromagnetic fields (RF-EMF): a pilot experimental study of personal exposure and risk perception

**DOI:** 10.1007/s40201-021-00636-7

**Published:** 2021-03-23

**Authors:** Berihun M. Zeleke, Christopher Brzozek, Chhavi R. Bhatt, Michael J. Abramson, Frederik Freudenstein, Rodney J. Croft, Peter Wiedemann, Geza Benke

**Affiliations:** 1 Centre for Population Health Research on Electromagnetic Energy (PRESEE), School of Public Health and Preventive Medicine, Monash University, Melbourne, VIC 3004 Australia; 2 Assessment and Advice Section, Radiation Health Services Branch, Australian Radiation Protection and Nuclear Safety Agency, 619 Lower Plenty Road, Yallambie, 3085 Australia; 3 Australian Centre for Electromagnetic Bioeffects Research, Illawarra Health and Medical Research Institute, School of Psychology, University of Wollongong, Northfields Ave, Wollongong, NSW 2522 Australia; 4 Department of Risk Communication, German Federal Institute for Risk Assessment, Max-Dohrn-Straße 8-10, 10589 Berlin, Germany

**Keywords:** Risk perception, Radiofrequency-electromagnetic fields, Personal exposure, Wi-fi

## Abstract

The impact of providing people with an objectively measured personal radiofrequency electromagnetic fields (RF-EMF) exposure information on the risk perception of people is not well understood. We conducted an experimental study, among three groups of participants, to investigate the risk perception of people towards RF-EMF from Wi-Fi sources (ISM 2.4 GHz) by providing participants with either basic text, precautionary information, or a summary of their personal RF-EMF exposure measurement levels. Participants provided with personal RF-EMF exposure measurement information were more confident in protecting themselves from RF-EMF exposure, compared to those provided with only basic information. Nonetheless, neither the exposure perception nor the risk perception of people to Wi-Fi related RF-EMF differed by the type of information provided. The measured Wi-Fi signal levels were far below international exposure limits. Furthermore, self-rated levels of personal RF-EMF exposure perception were not associated with objectively measured RF-EMF exposure levels. Providing people with objectively measured information may help them build confidence in protecting themselves from Wi-Fi related RF-EMF exposure.

## Introduction

Wi-Fi (Wireless-Fidelity) is a wireless network involving at least one Wi-Fi router connected to the Internet and a series of computers, laptops, smartphones, and/or other telecommunication devices using radiofrequency- electromagnetic fields (RF-EMF) [18]. As Wi-Fi exposure is becoming increasingly common, despite operating at low power, there is much concern about possible Wi-Fi health effects amongst the general public [2, 10, 17].

Many people perceive health risks from telecommunication sources and this includes concern about potential health effects associated with RF-EMF emissions from Wi-Fi in homes, workplaces, schools and other places [2, 9, 10, 15, 17, 19, 22].

In its 2010 research agenda for RF-EMF, the World Health Organization called for more studies to gain better understanding of RF-EMF and health, emphasizing the need to measure personal exposures in human epidemiological studies [25]. Nonetheless, previous studies that have assessed exposure and risk perception of people towards RF-EMF were subject to limitations due to either being questionnaire-based risk perception assessment without measuring personal exposure, or were limited to personal RF-EMF exposure measurements quantification without investigating risk perception [3, 4, 8, 24]. Although reporting higher risk perception levels may not be associated with higher levels of actual personal RF-EMF exposure [14], perceived exposure and health-risks due to RF-EMF are reportedly associated with higher symptom scores in the general population [20].

The characteristic RF-EMF emissions, exposure scenarios and corresponding exposure levels for wireless technologies have been addressed by a number of studies, and the levels of RF-EMF exposure from Wi-Fi sources have been reported to be far below major international limits [3, 4, 10, 30]. For instance, the Australian Radiation Protection and Nuclear Safety Agency (ARPANSA) conducted measurements of RF-EMF from Wi-Fi sources in classrooms and schoolyards and reported that the typical RF-EMF exposure from Wi-Fi sources was comparable or lower to other sources in the environment [17].

Recent research investigating RF-EMF exposure and risk perception found that individuals` knowledge of personal environmental RF-EMF exposure levels lowered their risk perception [23], and increased their confidence in protecting themselves from RF-EMF exposure [29]. Wi-Fi is one of the main contributors to personal RF-EMF exposure from environmental sources [19, 22, 23] and Wi-Fi related RF-EMF exposure has been an issue in the public debate [2, 17]. Nevertheless, epidemiological studies examining the health effects of Wi-Fi exposure are not available, as most of the RF-EMF epidemiological and measurement studies so far involved exposures to mobile (cellular) or cordless phones, and mobile phone base stations [2]. It is therefore important to measure the levels of exposure and to assess the levels of risk perception of people towards RF-EMF emitted from Wi-Fi sources. This will enable an understanding of the key factors that affect exposure perception and risk perception of people towards RF-EMF, and is crucial for developing adequate and informed risk communication strategies. In this experimental study, we objectively measured the RF-EMF from Wi-Fi sources (ISM 2.4 GHz downlink), and presented a summary of the RF-EMF exposure levels to a random group of participants. Furthermore, we compared their risk perception with participants who were provided with either basic information and/or precautionary messages regarding RF-EMF exposure.

This article is based on data from the same study that reported on risk perception of people to RF-EMF from mobile phone base station (MPBS) [29].). Although some of the frequency bands for Wi-Fi and MBPS overlap, most frequency bands for mobile phone use are lower (eg. 900 MHz). RF exposure from these RF-EMF sources are not similar in that MPBS are communal and uncontrollable, whereas Wi-Fi routers may be individually owned and could be controllable at times by switching off Wi-Fi routers. In addition, unlike for Wi-Fi routers, most people do not know where the nearest MPBS is located. Accordingly, it was reasonable to expect a different effect of exposure measurement information on risk perception of people from different sources of RF-EMF.

### Study hypothesis

This experiment study tested the hypothesis that people provided with objectively measured RF-EMF exposure levels from Wi-Fi sources have different levels of risk perception to those provided with only basic text or precautional information regarding RF-EMF.

## Methods

### Study sample

Based on a one-factor-experimental design, participants aged between 18 and 80 years were recruited and randomized into three groups. The details of the recruitment process and the data collection methods used have previously been published elsewhere [29, 30]. In brief, participants were invited to participate in the study via advertisements posted on notice boards at public libraries, universities, hospitals, and sporting clubs. After provided with an information pack detailing the study and consent forms, those who consented to participate in the study were then randomized to one of the three study groups (based on allocation ratios of 2:2:1): *1) the measurement group* (*n* = 63) who were provided with personal RF-EMF measurement devices that measured their RF-EMF exposure (ExpoM-RF), followed by the provision of a summary of their RF-EMF exposure levels from Wi-Fi sources; 2) *the precautionary group* (*n* = 158) who were provided with an information pack containing precautionary messages regarding RF-EMF which was similar to that provided by the Australian Radiation Protection and Nuclear Safety Agency (ARPANSA) [1]; and *3) the basic information group (n = 162)* who were only provided with basic information about RF-EMF. This same basic information about RF-EMF was also given to both the measurement and the precautionary information groups. Both the basic information and precautionary messages provided to participants were not limited to information on RF-EMF from Wi-Fi sources but regarding RF-EMF in general.

### Study procedures

#### RF-EMF exposure measurements

Personal RF-EMF exposure levels were measured with a portable ExpoM-RF device, developed by Fields At Work (GmbH, Zürich, Switzerland). The instructions for the personal measurements group were given at the time of handing the device to the subjects. The device measured electric field strengths in 16 frequency bands (87.5 MHz–5.8 GHz) including that emitted from Wi-Fi sources (ISM 2.4 GHz).

Each participant in the exposure measurement group carried an ExpoM-RF (with a sampling frequency of 10 s). On average, 9764 single measurements per participant were recorded over an average measurement time of 27.4 ± 4.5 h (range: 20.1–37.6 h) including time spent outside the home and night-time. During measurements, participants were instructed to continue their daily activities as usual while wearing the ExpoM-RF, and to place it on their bedside table or close to their bed during sleep. Subsequently, the average levels of personal exposure to Wi-Fi were calculated, which were then converted into proportions of the International Commission on Non-Ionizing Radiation Protection (ICNIRP) general public exposure limits [16]. The participants were provided with a summary of their personal exposure levels for the specified frequency band summarized as “personal RF-EMF exposure from Wi-Fi” together with a basic text information regarding RF-EMF exposure.

#### Exposure perception and risk perception assessment questionnaires

All participants were provided with a structured self-administered questionnaire that inquired various socio-demographic variables, exposure perception and risk perception to Wi-Fi related RF-EMF. Exposure perception was measured by asking “*To what extent do you think you are exposed to electromagnetic fields/radiation from Wi-Fi sources (on a scale of 1–7, where 1 = not at all; and 7 = very much)*?”. Risk perception of participants in the three groups to RF-EMF emissions from Wi-Fi sources was assessed in a similar fashion. Similarly, participants were asked to rate their degree of confidence in protecting themselves from RF-EMF emissions on a scale of 1-to-7 (*1 = not-at-all …. 7 = absolutely certain*). We specifically assessed on a Likert scale, how likely participants were to take preventive measures in order to protect themselves from the possible health-risks of RF-EMF from Wi-Fi sources by switching off wireless LAN routers at night or using a normal LAN instead of wireless LAN. In addition, participants were asked how frequently they thought or talked about the potential health effects of RF-EMF from Wi-Fi sources at home or at their workplace over the last 2 weeks.

#### Outcome variables

The outcome variables of this study were primarily related to the perception of exposure and risk among participants in each of the study groups, considering RF-EMF emitted from Wi-Fi sources. Hence, we considered the perception that the participants have of exposure levels (hereafter “exposure perception”), their perception of the effects of such exposure on their health (hereafter “risk perception”), their confidence in protecting themselves from RF-EMF (hereafter “confidence in protecting self”), and how likely participants would be going to take preventive measures to protect themselves from the perceived risks of RF-EMF exposure (hereafter, “prevention measures”).

#### Data analysis

Descriptive statistics are presented as frequencies, percentages, mean (SDs) and ranges. The RF-EMF exposures from the ISM 2.4 GHz frequency band were reported in electric field strength values (in V/m). For Wi-Fi, the ICNIRP reference limit is 1 × 10^6^ μW/m^2^ [16]. The normality of the geometric mean data of the personal RF-EMF measurements results were examined by Shapiro-Wilk tests of both untransformed and log-transformed data. For comparison with other studies, we converted the measured units into μW/m^2^.

Unadjusted tests of associations between outcome variables and each determinant variable were investigated using independent sample t-tests, one-way ANOVA or linear regression models. A separate analysis by means of a post hoc test (Tukey HSD) was performed if ANOVA was significant. Correlations between exposure perception and risk perception, as well as, perceived levels of exposure and measured exposure were assessed. Furthermore, adjusted analyses were performed using multiple linear regression models. All selected variables were fitted into the multiple linear regression model simultaneously using of the “enter” method. All analyses were performed using STATA version 15.0 (StataCorp, College Station, TX, USA). For all statistical tests, *p* value <0.05 (two sided) was used as a cut-off to declare statistically significant associations.

#### Ethics approval

Ethics was approved by Monash University Human Research Ethics Committee (MUHREC: Project Number: 8965) and written consent was obtained from each participant. Each participant was given AU $25 voucher upon completion as a reimbursement for their time during the survey.

## Results

### Characteristics of the study participants

A total of 383 people (58% women and 42% men) with mean (SDs) age of 34.3 (12.2) years participated in this study. By occupation, 136 (35.5%) were working or studying in academic and/or research environments, 96 (25.1%) office support and administration staff, 58 (15.1%) healthcare workers, and 54 (14.1%) were working in service industry. Overall, 95.3% lived in a house with a Wi-Fi router in place and 61.5% owned a Wi-Fi-enabled smart TV at home. Overall, there was no significant difference between the experimental study groups, in their demographic profile. The details of the socio-demographic profile of participants by study groups is presented in Table 1.

**Table 1:** Socio-demographic characteristics of participants

Characteristics	Total sample (*n* = 383)	Group			*p* value
		Basic information (*n* = 162)	Precautionary information (*n* = 158)	Dosimetry (*n* = 63)	
Age, mean (±SD) years	34.3±12.2	34.1±12.0	33.6±12.3	36.9±12.5	0.296
<30	172 (44.9)	80 (49.4)	71(44.9)	21(33.3)	
30–44	140 (36.6)	54 (33.3)	59 (37.3)	27 (42.9)	
45+	71 (18.5)	28 (17.3)	28 (17.7)	15 (23.8)	
Sex					
Female	222 (58.0)	94 (58.0)	86 (54.4)	42 (66.7)	0.251
Male	171(42.0)	68 (42.0)	72 (45.6)	21(33.3)	
Race/Ethnicity					
Caucasian (White)	217 (56.6)	93 (57.4)	91 (57.6)	33 (52.4)	0.091
Asian	90 (23.5)	32 (19.8)	35 (22.2)	23 (36.5)	
Others	76 (19.9)	37 (22.8)	32 (21.2)	7 (11.1)	
Education					
High school or less	93 (24.3)	43 (26.5)	40 (25.3)	10 (15.9)	0.305
Vocational training	78 (20.4)	27 (12.7)	36 (22.8)	15(23.8)	
University degree	212 (55.3)	92 (56.8)	82 (51.9)	38 (60.3)	
Occupation					
Service sector	54 (14.1)	27 (16.7)	22 (13.9)	5 (7.9)	0.125
Admin & Finance	96 (25.1)	44 (27.2)	36 (22.8)	16 (25.4)	
Healthcare worker	58 (15.1)	18 (11.1)	23 (14.6)	17 (27.0)	
Education/Researcher	136 (35.5)	59 (36.4)	57 (36.1)	20 (31.8)	
Other	39 (10.2)	14 (8.6)	20 (12.7)	5 (7.9)	
Wi-Fi router at home, yes	364 (95.3)	150 (92.6)	153 (96.8)	62 (96.8)	0.168
Wi-Fi enabled smart TV at home, yes	225 (58.9)	93 (57.4)	94 (59.5)	38 (61.3)	0.853

### Personal measurements

**Fig. 1:**
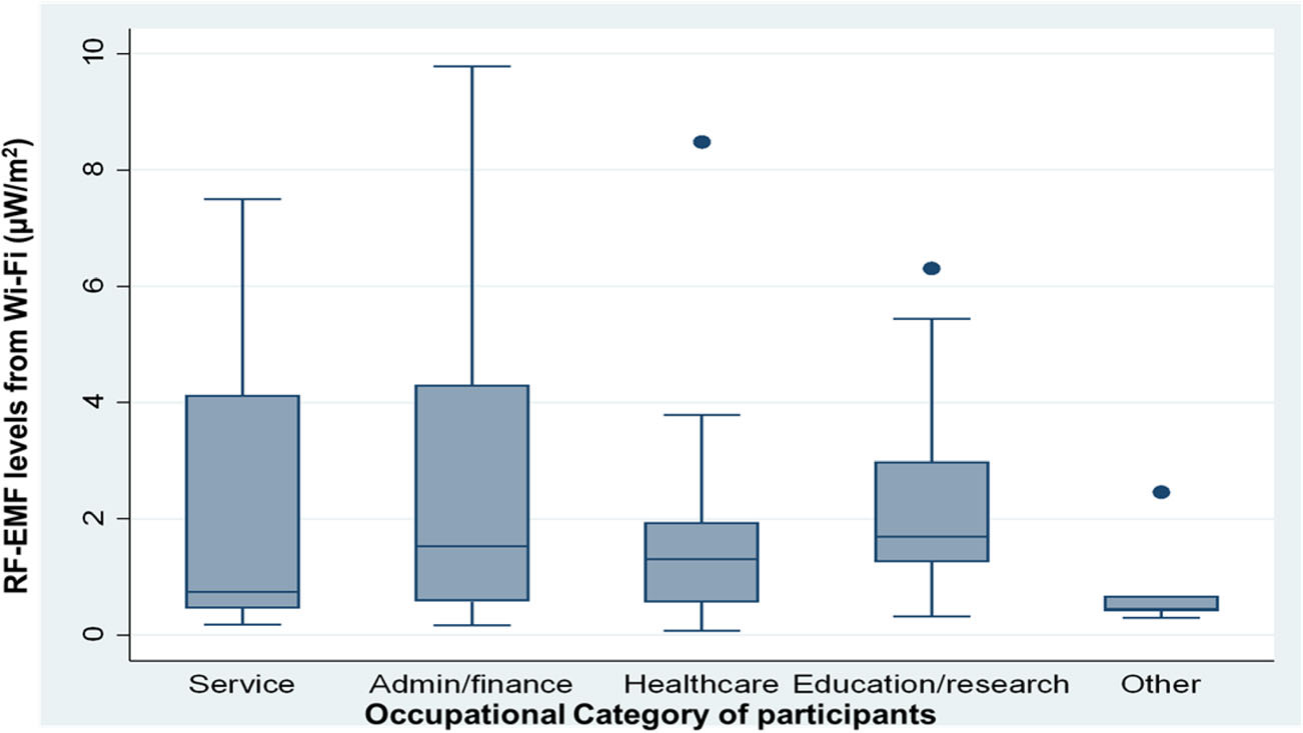
Median RF-EMF exposure to Wi-Fi sources (ISM 2.4GHz) by occupational category of participants

The median (25th & 75th percentiles) total RF-EMF exposure from Wi-Fi sources was 1.45 (0.63,3.23) μW/m^2^. As presented in Fig. 1, RF-EMF exposure levels (median; 25th & 75th percentiles) from Wi-Fi sources by occupation categories considered were ranked as: those who were working in the higher education and research sector (1.70; 1.25, 2.99 μW/m^2^), followed by office workers (1.53; 0.558,1.94 μW/m^2^), and those working in the healthcare sector (1.30; 0.55,1.94 μW/m^2^).

### Exposure and risk perception levels

In general, exposure perception to RF-EMF from Wi-Fi sources was moderately correlated (ρ = 0.564; *p* < 0.001) with risk perception. These correlations varied by study groups although the difference was not statistically significant (ρ = 0.635; p < 0.001 for the basic information, ρ = 0.599; p < 0.001 for precautionary, and ρ = 0.468; p < 0.001 for the personal measurement group) (Table 2). For the measurement group, measured personal RF-EMF exposure levels from Wi-Fi sources were poorly correlated with the participants perceived level of RF-EMF exposure (ρ = 0.168; *p* < 0.01).

**Table 2:** Spearman’s correlation matrix (ρ) between exposure perception, risk perception, prevention measures, and level of confidence in protection, by study groups

	Total sample (n = 383)				Study Group											
					Basic information (n = 162)				Precautionary information (n = 158)				Measured exposure information(n = 63)			
	Exposure perception	Risk perception	Prevention measures	Confidence in protecting self	Exposure	Risk perception	Prevention measures	Confidence in protecting self	Exposure perception	Risk perception	Prevention measure	Confidence in protecting self	Exposure perception	Risk perception	Prevention measure	Confidence in protecting self
Exposure Perception		**0.588**	**0.337**	**0.124**		**0.635**	**0.346**	0.089		**0.599**	**0.325**	**0.243**		**0.468**	**0.329**	−0.066
Risk Perception	**0.588**		**0.455**	**0.110**	**0.635**		**0.507**	0.108	**0.599**		**0.325**	**0.157**	**0.468**		**0.560**	0.002
Prevention measures	**0.337**	**0.455**		**0.155**	**0.346**	**0.507**		0.115	**0.325**	**0.325**		0.145	**0.329**	**0.560**		**0.253**
Confidence in protecting self	**0.124**	**0.110**	**0.155**		0.089	0.108	0.115		**0.243**	**0.157**	0.145		−0.066	0.002	**0.253**	

The numbers in bold is the statiscal significance of *p* < 0.05

**Fig. 2:**
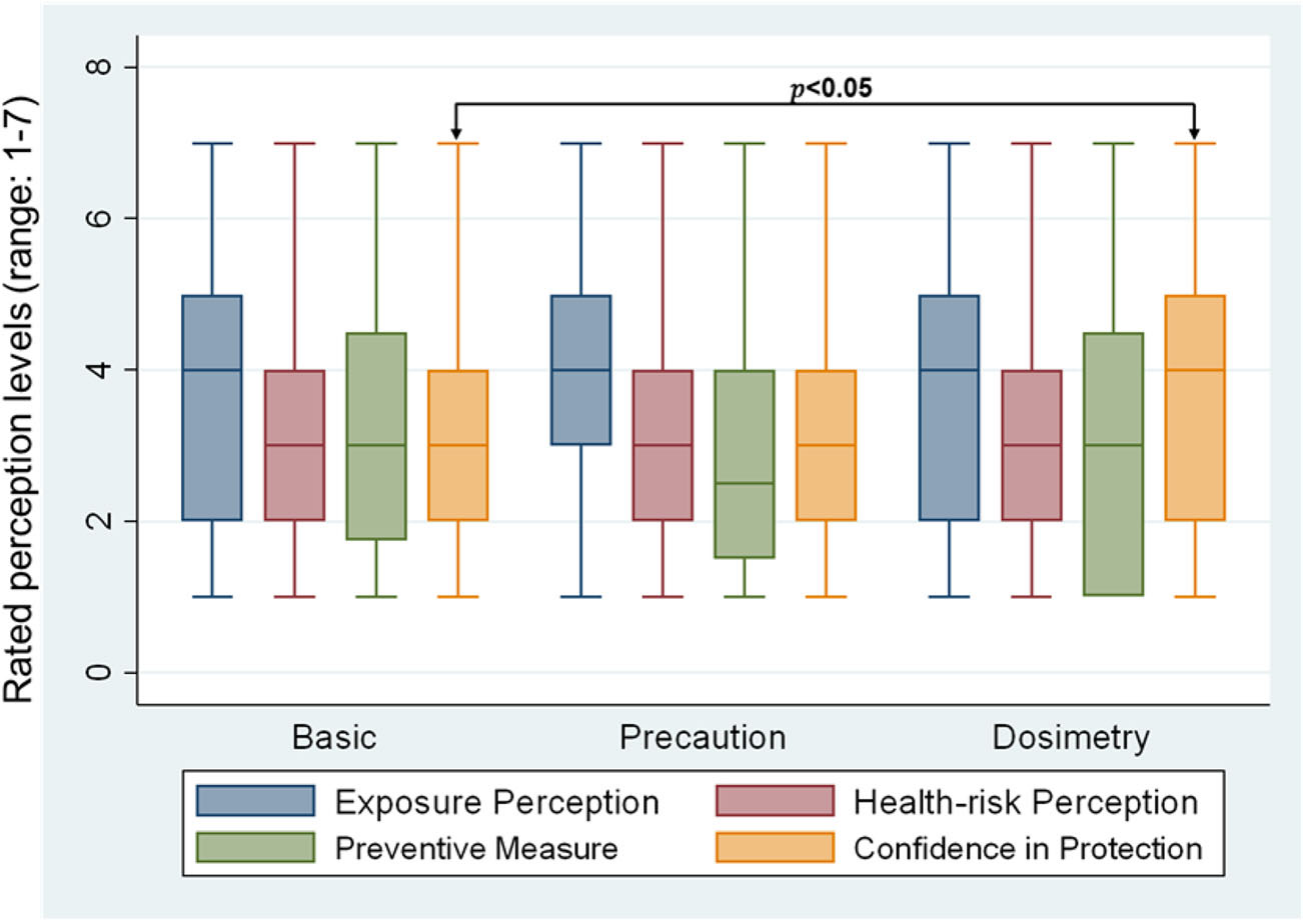
Study participants **e**xposure perception, risk perception, prevention measures taken, and confidence in protecting themselves from Wi-Fi related RF-EMF (*e. g., Question: on a scale of 1–7: 1 “not at all” to 7 “to a very dangerous”, how dangerous do you think are electromagnetic radiation emissions from Wi-Fi sources?”*

Figure 2 presents the median values (from a range of 1–7) for the exposure and risk perception of participants, prevention measures, and confidence in protecting themselves from RF-EMF related to Wi-Fi sources, by study groups. The personal RF-EMF exposure measurement group were more confident in being able to protect themselves from RF-EMF (β-coefficient = 0.551; *p* < 0.05) compared to those provided with only basic text. Nevertheless, the three groups did not significantly differ from each other in relation to their exposure perception or risk perception to RF-EMF emitted from Wi-Fi sources. Overall, over a third of the participants claim to either switch off wireless LAN router at home at night (38.6%) or use a normal LAN instead of wireless LAN (32.1%), over most of the time, to minimize their exposure. However, these measures were not significantly different among the three groups.

### Exploratory findings

In Table 3, linear regression models are presented for each of the outcome variables considered (exposure perception, risk perception, prevention measures, and confidence in protecting themselves from Wi-Fi related RF-EMF). The mean scores for Wi-Fi related RF-EMF exposure perception, risk perception or the likelihood of taking prevention measures did not significantly differ by experimental group. Nonetheless, the “personal exposure measurement” group reported higher mean scores for being confident in protecting themselves from Wi-Fi related RF-EMF compared to those provided with basic information groups (**β** = 0.551; *p* = 0.015) (Table 3). A separate post hoc analysis confirmed that the “personal exposure measurement” group had a statistically higher level of confidence in protecting themselves from RF-EMF (Bonferroni corrected *p* = 0.019) than those provided with only basic test of precautionary information (Bonferroni corrected *p* = 0.045).

**Table 3:** Linear regression models of selected variables and outcomes considered (exposure perception, risk perception, prevention measures, and confidence in protection) related to RF-EMF emitted from Wi-Fi sources

	Un-adjusted Analysis								Adjusted Analysis							
Variables	Exposure perception		Risk perception		Prevention measures		Confidence in self protection		Exposure perception		Risk perception		Prevention measures		Confidence in protecting self	
	β	p value	β	p value	β	p value	β	p value	β	p value	β	p value	β	p value	β	p value
Experimental group																
Precautionary vs. Basic information	0.123	0.584	−0.191	0.253	−0.259	0.174	−0.071	0.676	0.235	0.152	−0.161	0.205	−0.227	0.180	−0.046	0.786
Personal measurement vs. Basic information	−0.004	0.989	−0.111	0.615	−0.084	0.738	**0.551**	**0.015**	0.057	0.741	−0.217	0.207	−0.188	0.411	**0.632**	**0.006**
Age (years)	0.008	0.260	**0.016**	**0.012**	0.007	0236	**−0.014**	**0.024**	0.005	0.513	0.009	0.080	0.008	0.230	**−0.018**	0.010
Sex																
Female vs. Male	−0.073	0.693	**0.409**	**0.008**	0.001	0.994	−0.110	0.487	−0.251	0.116	**0.380**	**0.002**	−0.157	0.341	−0.154	0.353
Educational status																
Beyond high school vs. High school or less	−0.259	0.155	−0.017	0.914	−0.144	0.409	−0.063	0.692	−0.292	0.068	0.090	0.467	−0.279	0.091	−0.042	0.798
Ethnicity																
Caucasian vs. non-Caucasian	**−0.505**	**0.006**	**−0.427**	**0.005**	**−0.744**	**<0.001**	−0.161	0.311	−0.155	0.356	−0.186	0.151	**−0.597**	**<0.001**	0.098	0.572
Own a Wi-Fi enabled TV																
Yes vs. No	**−**0.051	0.781	0.176	0.255	0.062	0.725	0.099	0.533	−0.164	0.286	0.167	0.159	−0.064	0.686	0.090	0.570
Occupation																
Admin & Finance	Ref	Ref	Ref	Ref	Ref	Ref	Ref	Ref	Ref	Ref	Ref	Ref	Ref	Ref	Ref	Ref
Service sector	0.116	0.969	**−0.507**	**0.043**	−0.266	0.362	−0.093	0.724	0.262	0.310	−0.318	0.112	−0.242	0.363	−0.202	0.450
Healthcare worker	0.254	0.390	0.254	0.299	0.430	0.127	−0.047	0.873	0.111	0.659	0.019	0.922	0.320	0.217	−0.226	0.386
Education/Researcher	0.271	0.252	−0.310	0.113	−0.094	0.677	0.345	0.164	**0.498**	**0.013**	**−0.325**	**0.037**	−0.044	0.833	0.172	0.409
Other	0.604	0.073	0.386	0.166	0.247	0.442	0.131	0.685	0.214	0.445	0.181	0.405	−0.124	0.668	−0.026	0.928
Exposure perception	–	**–**	**0.474**	**<0.001**	**0.310**	**<0.001**	0.082	0.063	–	**–**	**0.384**	**<0.001**	0.076	0.164	0.050	0.362
Risk perception	**0.672**	**<0.001**	–	–	**0.504**	**<0.001**	0.076	0.149	**0.646**	**<0.001**	–	–	**0.370**	**<0.001**	0.042	0.553
Prevention measures	**0.338**	**<0.001**	**0.385**	**<0.001**	*–*	*–*	***0.115***	***0.013***	0.078	0.120	**0.216**	**<0.001**	–	–	0.087	0.100
Discussion about risks of Wi-Fi with a friend/relative	**−1.101**	**<0.001**	**−1.154**	**<0.001**	**−1.261**	**<0.001**	−0.328	0.188	−0.289	0.733	**−0.409**	**0.019**	**−0.727**	**0.004**	−0.044	0.864

(β-coefficients and *p* values as presented for unadjusted and adjusted analysis; statistical significance set at *p* < 0.05)

After adjusting for potential confounders (gender, age, ethnicity, education, and ownership of Wi-Fi enabled devices), some significant associations were identified. Participants who perceived risk from Wi-Fi related RF-EMF were more likely to perceive higher RF-EMF exposure (**β** = 0.646; *p* <0.001). Women were more likely to perceive risk (**β** = 0.380; *p* <0.01) to RF-EMF emitted from Wi-Fi sources compared to men. Older participants had lower mean scores (**β** = −0.018; *p* <0.001) for their confidence in protecting themselves from RF-EMF while participants who were provided with their RF-EMF personal exposure from Wi-Fi sources reported higher scores for confidence (**β** = 0.632; *p* <0.01) (Table 3). Furthermore, the adjusted analysis demonstrated that participants who identified themselves as Caucasian (**β** = −0.597; *p* <0.001) were less likely to consider taking prevention measures such as switching off wireless routers at night. We also observed that if people perceived more risk, they are more likely to take preventive measures, irrespective of the level of exposure information provided. Similarly, participants who discussed the possible health-risks of RF-EMF with a friend/relative during the preceding fortnight were more likely to have lower risk perception scores (**β** = −0.409; *p* <0.05), and are less likely to take preventive measures (**β** = −0.727; *p* <0.01).

## Discussion

In this experimental study, participants were randomized into three groups. Each group was provided with different type of RF-EMF exposure information: (1) basic information, (2) precautionary information or (3) information about one’s own exposure level. Subsequently, participants were asked to rate their confidence in protecting themselves, their level of exposure perception and risk perception, and the prevention measures they would take in the future.

Participants who were provided with RF-EMF personal exposure measurement information were more confident about protecting themselves from RF-EMF exposure. However, the three experiment groups did not significantly differ from each other in relation to their exposure perception, risk perception, and the prevention measures they are likely to take.

Previous studies reported increased risk perception after the provision of precautionary information and selected media reports that strongly suggested the harmfulness of EMFs [5, 26, 28]. In contrast, we hypothesised that risk perception would be lower after receiving objectively measured personal RF-EMF exposure from Wi-Fi sources compared to the provision of precautionary or basic text information. Similarly, Gallastegi M and colleagues [14], recently reported higher perception levels were not associated with higher levels of measured RF-EMF from Wi-Fi sources. It was reassuring that participants in the personal measurement group have had higher levels of confidence in protecting themselves from Wi-Fi related RF-EMF exposure. Since participants in the personal measurement group were made aware that their exposure was negligible and hence their exposure perception was low, while the other two groups may still have a higher exposure perception that was positively correlated to their confidence in protecting themselves from RF-EMF exposure. With the ever-increasing RF-EMF exposures in human populations, if the goal is to identify possible health effects of RF-EMF exposures from Wi-Fi technology, it would be important to objectively quantify the exposure levels of the subjects, as we have done in this study.

Previous studies have also demonstrated that indoor RF-EMF exposure is increasing faster than outdoor exposure because of widespread use of home wireless devices and short-range communication systems [13]. In the current study, the study groups were not significantly different in their risk perception to Wi-Fi exposure. The exposure levels found in our study for ISM 2.4 GHz (RF-EMF emitted from Wi-Fi sources) were all well-below the reference levels (< 1%) for the general public as provided in the guidelines of the ICNIRP and the ARPANSA [1, 16], but similar to those reported by previous studies including one conducted in Melbourne, Australia [3, 4, 8]. The low levels of RF-EMF exposure measured from Wi-Fi sources may partly explain the low risk perception and high confidence in protection findings in this study.

Although such provisions specific to RF-EMF from Wi-Fi sources were not investigated in previous studies, provision of precautionary messages was observed to reduce trust in sufficient protection from the health effects of RF-EMF and that information may increase concern [21, 27]. Nevertheless, our findings demonstrated that the provision of objectively measured personal RF-EMF exposure levels from Wi-Fi sources showed no significant difference in the risk perception, nor in prevention measures, compared to basic or precautionary measure information provision. This may partly due to the operationalisation of the dependent variables and a more finely granulated scales for both exposure and risk perception would be able to catch differences among the experimental groups. Exposure perception to Wi-Fi related RF-EMF and risk perception were moderately correlated. This is consistent with previous research that reported the correlation between perceived exposure and risk perception to RF-EMF in general [6, 11, 12], as well as that from Wireless Local Area Networks (WLAN) [5].

This study is unique in that it was an experimental design, the measurement and provision of objectively assessed personal RF-EMF exposure to participants, and the assessment of risk perceptions were carried out among a random sample recruited from the general public. In former studies, risk perception was directly assessed after some information provision had been given [5, 7, 21, 26], but without objectively measured exposure information. For the current study, both exposure and risk perception to Wi-Fi related RF-EMF were directly connected to their objectively measured personal RF-EMF exposure levels. RF-EMF measurement devices (ExpoM-RF) are one of the best current tools for personal RF-EMF exposure [24] and personal exposure measurements were conducted over a 24 h period, allowing for a description of RF-EMF exposure during all hours of the day [30]. Nevertheless, caution should be taken while interpreting the measurement results since on-body calibration or assessment of inter-sample variation between measurement devices was not performed for the current study.

Our findings could be limited by the relatively small sample size, specifically in the personal exposure measurement group, making it difficult to investigate within group differences and inability to perform multiple comparisons. Although random allocation was performed, participants were recruited via advertisements and may be pre-selected based on their access to the invitations and voluntary expression of their interest for participation and hence may not be representative of the source population. However, this study, being a pilot experiment, provides a basis for future research with greater statistical power, which could provide a more comprehensive investigation of the effects of providing Wi-Fi related personal RF-EMF exposure measurement information, and subsequent assessment of risk perception.

## Conclusions

There was no difference between the experimental groups in their exposure perception, or risk perception to Wi-Fi, and the prevention measures likely to be taken. However, it cannot be ruled out that this result is due to chance. Nonetheless, our data indicated that the participants in the measurement group were more confident in being able to protect themselves from Wi-Fi related RF-EMF exposure. Therefore, providing people with objectively measured exposure data may still be a useful option for supporting evidence-based judgements on Wi-Fi related RF-EMF exposure and associated risks. Further studies are needed to get more robust data on the impact of RF-EMF measurement information on exposure and risk perceptions to Wi-Fi related RF-EMF sources.

## Abbreviations

RF-EMF: Radiofrequency-Electromagnetic Field
Wi-Fi: Wireless-Fidelity
